# KAT8 catalyzes the acetylation of SEPP1 at lysine 247/249 and modulates the activity of CD8^+^ T cells via LRP8 to promote anti-tumor immunity in pancreatic cancer

**DOI:** 10.1186/s13578-025-01356-3

**Published:** 2025-02-19

**Authors:** Zhongfei Zhu, Gang Nie, Xiaobo Peng, Xianbao Zhan, Dan Ding

**Affiliations:** 1 Department of Hepatobiliary, Pancreatic and Spleen Surgery, The First Affiliated Hospital of Naval Medical University, Shanghai, 200433 China; 2 Department of Oncology, The First Affiliated Hospital of Naval Medical University, No. 168, Changhai Road, Yangpu District, Shanghai, 200433 China; 3 Department of Gastrointestinal Surgery, The First Affiliated Hospital of Naval Medical University, No. 168, Changhai Road, Yangpu District, Shanghai, 200433 China

**Keywords:** SEPP1, LRP8, Immune escape, Pancreatic cancer

## Abstract

**Background:**

Pancreatic cancer (PC) remains one of the most lethal malignancies with unfavorable prognosis globally. Bioinformatics analysis predicted that SEPP1 was low expressed in PC and related to tumor immune microenvironment, but its biological function was still unclear.

**Methods:**

PC xenograft and liver metastasis mouse models, as well as PC cell-MDSCs co-culture system, were established for in vivo and in vitro studies, respectively. The expression and localization of key molecules were detected by qRT-PCR, western blot, immunohistochemistry and immunofluorescence. Flow cytometry was employed to assess the abundance of immune cells and cell apoptosis. The interactions among KAT8, SEPP1 and LRP8 were detected by co-IP. Cell viability, migration and invasion were monitored by CCK-8 and transwell assays.

**Results:**

SEPP1 was downregulated in pancreatic tumors, and it was positively correlated with the abundance of CD8^+^ T cells. In vivo overexpression of SEPP1 impaired PC tumor growth and liver metastasis via modulating the abundance of CD8^+^ T cell and MDSCs. KAT8 upregulated SEPP1 transcription and protein level via catalyzing the acetylation at K247/249 on SEPP1, and SEPP1 impaired MDSCs survival via its receptor LRP8, thus regulating CD8^+^ T cell-mediated immune responses in PC. In vivo studies further revealed that SEPP1 recombinant protein enhanced the efficacy of anti-PD-1 therapy in PC xenograft mouse model.

**Conclusion:**

KAT8 catalyzed the acetylation of SEPP1 at K247/249 and modulated the activity of CD8^+^ T cells via LRP8 to promote anti-tumor immunity in PC.

**Supplementary Information:**

The online version contains supplementary material available at 10.1186/s13578-025-01356-3.

## Highlights

Downregulated SEPP1 in pancreatic tumors was positively associated with the abundance of CD8^+^ T cells.

KAT8 upregulated SEPP1 protein level through catalyzing the acetylation at K247/249 on SEPP1.

KAT8 suppressed PC progression via modulating SEPP1-dependent immune responses.

SEPP1 impaired MDSCs survival in a LRP8-dependent manner.

SEPP1 recombinant protein enhanced the efficacy of anti-PD-1 therapy in PC mouse model.

**Supplementary Information:**

The online version contains supplementary material available at 10.1186/s13578-025-01356-3.

## Introduction

Pancreatic cancer (PC) remains one of the most lethal malignancies worldwide, and it has unfavorable prognosis and dismal 5-year survival due to lack of early detection method and difficulty in treatment [1]. Given the increased lifespan and PC-specific risks, the number of newly diagnosed PC patients was increasing rapidly during the past two decades [2]. It is well-established that tumor microenvironment (TME) which consists of tumor cells, endothelial cells, stromal fibroblasts and immune cells plays a pivotal role in PC progression [3]. Previous studies have illustrated that PC tumors exhibit low filtration of CD8^+^ T cells, while accumulation of myeloid-derived suppressor cells (MDSCs) has been observed in tumors and metastases in PC model which contributes to the growth and metastasis of PC [3, 4]. In recent years, the therapeutic benefits of PD-1 therapy have been reported in various cancers [5], however, the majority of PC are resistant to single treatment with anti-PD-1 [6]. It is of interest to develop a combination therapy with anti-PD-1 for PC treatment.

Selenoprotein P (SEPP1), a member of selenoprotein family, is recognized as a selenium supply protein and antioxidant enzyme [7, 8]. SEPP1 is a secreted selenoprotein which is primarily synthesized in the liver, and the serum level and expression of SEPP1 correlate with the incidence, survival and aggressiveness of cancer [9, 10]. For instance, low SEPP1 level is associated with unfavorable prognosis and inversely correlated with tumor grade and stage in renal, colon and prostate cancers [10]. Accumulating evidence suggests that SEPP1 is downregulated and acts as a tumor suppressor in a variety of cancers, including prostate cancer, colorectal cancer (CRC), hepatocellular carcinoma (HCC) and gastric adenocarcinoma [11–15]. These reports mainly focused on the antioxidant function of SEPP1 in different cancers. For instance, downregulated SEPP1 increases free radicals and facilitates the progression of prostate cancer [12]. Overexpression of SEPP1 impairs HCC cell proliferation, accompanied with decreased ROS production [15]. Intriguingly, the analysis based on Tumor IMmune Estimation Resource (TIMER, https://cistrome.shinyapps.io/timer/) web database revealed that SEPP1 expression positively correlated with the abundance of CD8^+^ T cells, indicating its potential role in immune escape and anti-tumor immune response in PC.

LRP8, also known as ApoER2, is recognized as a receptor of SEPP1 in testis and brain [16, 17]. The interaction between SEPP1 and LRP8 promotes the uptake of SEPP1 by Sertoli cells, thereby maintaining the selenium level for spermatogenesis [17]. In addition, LRP8 knockout mice fed with low-selenium diet exhibit neurological dysfunction and death, indicating that SEPP1/LRP8 association also plays an indispensable role in maintaining brain selenium level and protects against neurological dysfunction [16]. However, the regulatory mechanisms underlying SEPP1-LRP8 axis-regulated selenium uptake in the testis and brain remain uninvestigated. More importantly, previous study has demonstrated that LXR/ApoE axis impairs the viability of MDSCs through LRP8, thus facilitating anti-tumor immunity by activating cytotoxic T lymphocytes (CTLs). LXR/ApoE fails to enhance apoptosis of LRP8-knockout MDSCs, indicating the indispensable role of LRP8 in LXR/ApoE-restricted innate immune suppression [18]. It has been reported that LRP8 is elevated in pancreatic tumors, and ApoE2-LRP8 facilitates cell cycle function to promote PC cell proliferation via ERK/c-Myc/p21^Waf1^ signaling [19]. Our preliminary bioinformatics analysis based on GPS-PAIL web database (http://pail.biocuckoo.org/) predicted the acetylation sites of SEPP1 at lysine 247/249 (K247/249), and histone acetyltransferase (HAT) KAT8 might be responsible for the lysine acetylation of SEPP1. We thus hypothesized that KAT8 catalyzed the acetylation of SEPP1 at K247/249, thereby regulating MDSCs survival via LRP8 and CD8^+^ T cells-mediated immune responses in PC.

In this study, we reported that downregulated SEPP1 in PC was positively associated with the abundance of CD8^+^ T cells. KAT8 upregulated SEPP1 protein level through catalyzing the acetylation at K247/249 on SEPP1, and SEPP1 promoted MDSCs survival via its receptor LRP8, thus regulating CD8^+^ T cell-mediated immune responses. These findings shed light on the combination therapy of SEPP1 recombinant protein and anti-PD-1 antibody for PC treatment.

## Materials and methods

### Public datasets and tumor immunity analyses

Differentially expressed genes (DEGs) were identified using GEO database. Four GEO public datasets were used in this study, including GSE183795, GSE19650, GSE125158 and GSE46234. The association between SEPP1 expression and the abundance of CD8^+^ T cells were analyzed using tumor immune estimation resource (TIMER, http://cistrome.shinyapps.io/timer/).

### Clinical specimens

A cohort of 34 patients with PC in The First Affiliated Hospital of Naval Medical University from 2021 to 2022 were recruited to this study. Pancreatic tumors and their adjacent normal tissues were harvested. This study was approved by the Ethic Committee of The First Affiliated Hospital of Naval Medical University (Changhai Hospital) (No. CHEC2019-216). Consents were obtained from all patients.

### Cell culture, treatment, transfection and co-culture system

Human pancreatic duct epithelial cell lines H6C7 and hTERT-HPNE cells, human PC cell lines PANC-1, BxPC-3, CFPAC-1 and Capan-2 cells, and HEK293T cells were from ATCC (Manassas, VA, USA). Mouse-derived PC cell line: KPC cells were derived from the pancreatic tumor of C57BL/6-*Trp53*^em4(R172H)^*Kras*^em4(LSL−G12D)^Tg(Pdx1-cre) Smoc mice. Pan02 cells were purchased from Procell Company in Wuhan, China. PANC-1, hTERT-HPNE and HEK293T cells were cultured in DMEM containing 10% FBS (Gibco, Grand Island, NY, USA). BxPC-3, CFPAC-1, Capan-2, KPC and Pan02 cells were grown in RPMI1640 supplemented with 10% FBS (Gibco), and H6C7 cells were cultured in Keratinocyte SFM containing 5 ng/mL EGF and 50 µg/mL bovine pituitary extract (Gibco). Cells were treated with 2 µM MG149 (Sigma-Aldrich, St. Louis, MO, USA) for 24 h, or 5 µg/mL cycloheximide (CHX, Sigma-Aldrich) for 0, 4, 8 h. Cells were treated with concentration KAT8 recombinant protein (rmKAT8, vendor) for 48 h. All cells were maintained at 37 °C/5% CO_2_. The full-length of SEPP1 or KAT8 was cloned into pcDNA3.1 vector. The K-to-R mutants were generated by PCR mutagenesis. For co-IP, the full-length of SEPP1 was cloned into pcDNA3.1-Flag vector (Sangon, Shanghai, China). Sh-NC, sh-LRP8-1, sh-LRP8-2, sh-SEPP1 and sh-KAT8 were purchased from Sangon (Shanghai, China). All transfections were conducted using Lipofectamine 3000 reagent (Invitrogen, Carlsbad, CA, USA). For the co-culture system, PANC-1 or BxPC-3 cells (5 × 10^5^ cells /mL) were resuspended and 200 µL of cell suspension were added to culture inserts in the lower (Corning, Corning, NY, USA) for culture, and co-cultured with the same number of MDSCs in upper chambers for 6 days.

### Animal study

Male BALB/c nude mice or C57BL/6 mice (6 ~ 8-week-old, *n* = 5 per group) were from Guangdong medical experimental animal center (Foshan, China). Stable transfected PANC-1 cells (4 × 10^6^ cells) were inoculated into the right axillary fossa of BALB/c nude mice. KPC and Pan02 cells (5 × 10^5^ cells) were injected into the flank of C57BL/6 mice subcutaneously as described [20, 21]. Tumor size was measured every 4 days, and tumor volume was calculated as 0.5×length×width^2^. For liver metastasis assay, stable transfected Pan02 cells (1 × 10^6^ cells) were injected into the tail vein of C57BL/6 mice as described [22]. On day 35 post-inoculation, mice were sacrificed and liver tissues were dissected for subsequent analysis. All animal studies were approved by the Ethic Committee of The First Affiliated Hospital of Naval Medical University (Changhai Hospital) (No. EHBHKY2022-H009-P001). MG149 (1 mg/kg/d, Sigma-Aldrich), monoclonal antibody against LRP8 (200 µg/mouse, custom-made at Sangon, Shanghai, China) and anti-PD-L1 antibody (10 mg/kg/mouse, 29 f.1a12; Bio X cells) or/and mouse SEPP1 recombinant protein (200 µg/mouse, custom-made at Sangon, Shanghai, China) were injected intraperitoneally every 4 days from the 15th day after tumor injection, with a total of 5 injections. To induced MDSC depletion, C57BL/6 tumor-bearing mice were treated with RGX-104 (100 mg/kg/mouse/day) or control diet for 3 days, or treated with GW3965 (100 mg/kg/mouse/day) or control diet for 4 days as described [18].

### Immunohistochemistry (IHC) analysis

Paraffin-embedded pancreatic tumors and their normal counterparts were cut into 5 μm-sections, followed by deparaffinization, rehydration and antigen retrieval. The sections were then blocked with 10% normal goat serum and incubated with anti-SEPP1 (1:100, ab155185, Abcam, Cambridge, UK) or anti-Ki-67 (1:100, ab15580, Abcam) at 4 °C overnight. The immunoreactivities were detected using anti-rabbit secondary antibody-HRP (31460, Invitrogen, Carlsbad, CA, USA) and DAB substrate (Beyotime, Haimen, China). For immunofluorescence (IF) detection, the slides were incubated with donkey anti-rabbit AlexaFluor Plus 555 secondary antibody (A32794, Invitrogen), and images were acquired using a confocal microscope (Nikon, Toyoko, Japan).

### In vivo bioluminescence imaging

PANC-1-luciferase cells were transfected and SEPP1 overexpression construct, and stable transfected PANC-1 cells (4 × 10^5^ cells) were obtained and injected. On 3, 7 and 17 day post-inoculation, the anesthetized mice were injected with 150 mg/kg D-luciferin (Invitrogen) intraperitoneally. After 10 min, images were acquired using IVIS in vivo imaging system (PerkinElmer, Waltham, MA, USA).

### Hematoxylin and eosin (H&E) staining

Paraffin-embedded sections were dewaxed, rehydrated and stained with H&E solution (Sigma-Aldrich). Images were acquired under a microscope (Nikon).

### Flow cytometry

Xenograft tumors were cut into minute pieces and digested using HBSS containing 1 mg/mL DNase I, 1 mg/mL Collagenase D and 2.5 mg/mL Hyaluronidase (Sigma-Aldrich), followed by the dissociation using a dissociator (Miltenyi Biotec, Bergish Gladback, Germany). After centrifugation and red blood cell removal, cells were resuspended in Flow cytometry staining buffer (Invitrogen) and stained with antibodies. Freshly harvested tumor cells were stained with antibodies against surface marker CD3 (ab16669, Abcam), CD4 (ab133616/ab183685, Abcam), CD8 (ab237709/ab217344, Abcam), CD11b (ab8878, Abcam), CD11c (ab219799, Abcam), CD15 (ab135377, Abcam), CD19 (ab245235, Abcam), CD49b (ab272261, Abcam), CXCR3 (12-1839-42/17-1831-82, Invitrogen), F4/80 (ab16911, Abcam), Ly-6G (ab238132, Abcam), MHC II (ab139365, Abcam) and LRP8 (MA5-38566, Invitrogen). Cells were then fixed and permeabilized using Intracellular Fixation & Permeabilization Buffer set (88-8824-00, Invitrogen), and stained with anti-IFN-γ antibody (12-7319-42/12-7311-82, Invitrogen). Cell apoptosis was assessed by Annexin V-FITC/PI staining using Annexin V apoptosis kit (Invitrogen). For the gating strategy, cells were stained with surface markers CD4 (ab133616) and CD8 (ab217344) antibodies. CD4-positive or CD8-positive cells were gated. Signals were analyzed using CytoFLEX SRT (Baker Corporation).

### Reverse transcription quantitative polymerase chain reaction (RT-qPCR)

Total RNA was extracted from cells or pancreatic tissues using Trizol (Invitrogen) and reverse transcribed using SuperScript III reverse transcriptase (Invitrogen). qRT-PCR was conducted using SYBR Green quantitative RT-qPCR kit (Sigma-Aldrich) with ABI7500 Reak-Time PCR System (ABI, Foster City, CA, USA), and analyzed using 2 ^–ΔΔCT^ method. Primers were listed in Table 1.

**Table 1:** The primer sequence

Primer	Sequence 5’-3’
hSEPP1-F	AACAGAGAGCCAGGACCAAA
hSEPP1-R	GCCTGCAGTATGCACAGGTA
mSEPP1-F	TGTTGAAGAAGCCATTAAGATCG
mSEPP1-R	CACAGTTTTACAGAAGTCTTCATCTTC
hKAT8-F	GAAGGAGCATGAGGCGATCA
hKAT8-R	TTTCGTAGTTCCCGATGTGGAT
mKAT8-F	ACGAGGCGATCACCAAAGTG
mKAT8-R	AAGCGGTAGCTCTTCTCGAAC
hFLT3-F	AAGCAATTTAGGTATGAAAGCCAGC
hFLT3-R	CTTTCAGCATTTTGACGGCAACC
hSTEAP4-F	CACTGCTCGCCTTGGTTTA
hSTEAP4-R	TTGCCTGGGTAGCGGTTCT
hRGS2-F	CCTCTACTCCTGGGAAGCCC
hRGS2-R	CCTGAATGCAGCAAGACCATATTT
hIFRD1-F	ATCGGACTGTTCAACCTTTCAG
hIFRD1-R	GCACTCTTATCAAGGGTTAGGTC
hGRB10-F	AGGACACAGCACTGGTTTCACG
hGRB10-R	TCTGGCTGTCACGGAGGAGAAA
hLINC00671-F	GAATTGAAGTGTGGTTCCCAC
hLINC00671-R	TGTCTTTCCTGGGAGTGAGAT
hPRKAR2B-F	TTCGGCGAACTGGCCTTAATG
hPRKAR2B-R	ACTTTCAGGCGTTCAGAAAACT
hBEX1-F	GAGAACCACAGGCAAGGATG
hBEX1-R	CAGGGCATAAGGCAAAACTC
hCD36-F	GCCAGTTGGAGACCTGCTTA
hCD36-R	CTCAGCGTCCTGGGTTACAT
hLYVE1-F	CAGCACACTAGCCTGGTGTTA
hLYVE1-R	CGCCCATGATTCTGCATGTAGA
hPDK4-F	CAAGATGCCTTTGAGTGTTCAA
hPDK4-R	GGTCTTCTTTTCCCAAGACAAC
hERO1B-F	TGACAAAAAGGGGGCCAAGT
hERO1B-R	TATCGCACCCAACACAGTCC
hSERPINI2-F	GAGCCAAAGGAAAAGCACAG
hSERPINI2-R	TGCATCTTGAAAATCCACCA
hGAPDH-F	GAGTCCACTGGCGTCTTCA
hGAPDH-R	GGTCATGAGTCCTTCCACGA
mGAPDH-F	AGCCCAAGATGCCCTTCAGT
mGAPDH-R	CCGTGTTCCTACCCCCAATG

### Western blot

Protein lysates were extracted using RIPA lysis buffer (Beyotime). Equal amount proteins were separated by gel electrophoresis, and transferred onto PVDF membrane (Beyotime). After blocking with 5% non-fat milk, the blots were incubated with anti-KAT8 (1:1000, ab200660, Abcam), anti-SEPP1 (1:1000, ab155185, Abcam), anti-acetyl lysine (1:1000, ab190479, Abcam), anti-Myc tag (1:1000, ab32, Abcam) or anti-β-actin (1:2000, ab8226, Abcam) antibody at 4 °C overnight. This is followed by the incubation with secondary antibody-HRP (31460 or 31430, Invitrogen). Signal was detected using ECL substrate (Beyotime).

### Immunofluorescence (IF)

HEK293T cells or MDSCs were fixed in 4% paraformaldehyde (PFA) and permeabilized with 0.1% Triton X-100. After blocking with 1% BSA, cells were then incubated with anti-KAT8 (1:200, ab200660, Abcam), anti-SEPP1 (1:50, ab277526, Abcam) or anti-LRP8 (1:100, PA5-87962, Invitrogen) antibody at 4 °C overnight. This is followed by the incubation with Alexa Fluor 488-conjugated secondary antibody (1:500, A21428, Invitrogen). Images were acquired using a confocal microscope (Nikon).

### Co-immunoprecipitation (co-IP)

Cell lysates were extracted using IP lysis buffer (Pierce, Rockford, IL, USA). Proteins were then incubated with anti-KAT8 (1:170, ab200660, Abcam), ant-SEPP1 (1:30, ab277526, Abcam), anti-Flag tag (1:30, ab205606, Abcam) antibody or normal IgG at 4 °C overnight. This is followed by the incubation with Protein A/G agarose (Pierce), and the elutes were analyzed by western blot.

### Cell viability assay

PANC-1 and BxPC-3 cells were seeded in 96-well plates 24 h prior to transfection. At designated time points, cells were incubated with 10 µL/well CCK-8 solution (Beyotime, ) at 37 °C for 1 h. A450 was measured using a microplate reader (Bio-Rad, Hercules, CA, USA).

### Transwell assay

PANC-1 and BxPC-3 cells were plated onto Matrigel-coated culture inserts (Corning) and cultured in serum-free medium. The lower chambers were filled with complete medium. After 1 day, the invading cells were fixed and stained with crystal violet, and images were acquired under a microscope (Nikon). Transwell migration assay was performed by the similar approach without Matrigel coating.

### Cytotoxic T lymphocyte (CTL) assay

CD8^+^ T cells were isolated from the spleen of C57BL/6 mice and incubated with tumor cells (PANC-1 or BxPC-3) for 3 days. The cytolytic activity of PANC-1 or BxPC-3 cells (effector cells: target cells = 50: 1) was then tested by using lactate dehydrogenase (LDH) assay kit (ab102526, Abcam).

### Statistical analysis

All experiments were conducted at least 3 times, and data were presented as means ± S.D. Data were analyzed using GraphPad 8.0. One-way ANOVA or two-tailed Student’s *t*-test was used to analyze the multi-group or two-group comparison, respectively. *P* < 0.05 was considered statistically significant.

**Fig. 1:**
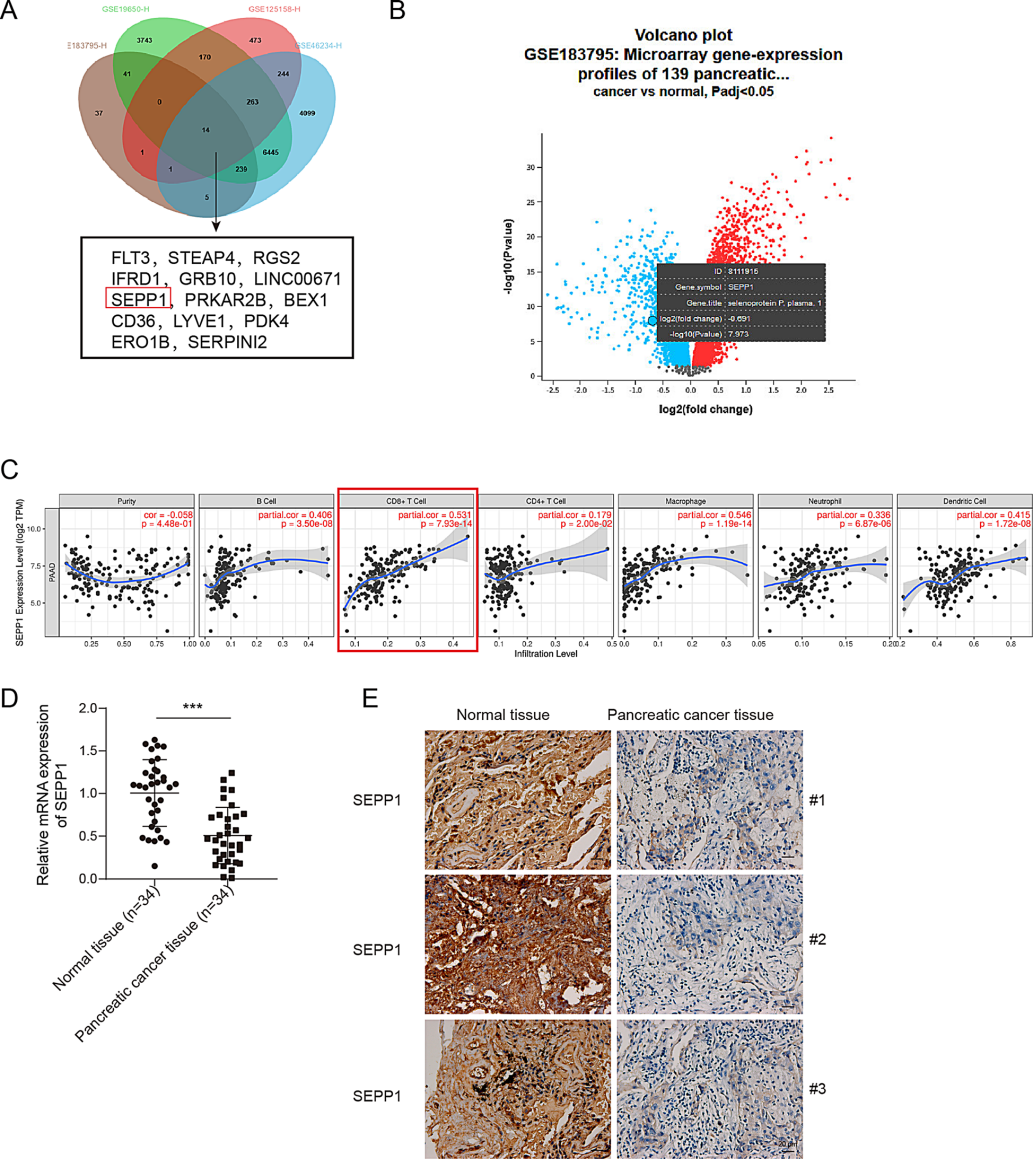
SEPP1 is downregulated in pancreatic tumors, and it is associated with the abundance of CD8^+^ T cells. (**A**) DEGs were identified by GEO datasets (GSE183795, GSE19650, GSE125158 and GSE46234). (**B**) Venn diagram comparing DEGs identified by GEO datasets. (**C**) The association between SEPP1 expression and the abundance of CD8^+^ T cells were analyzed using TIMER. (**D**) The mRNA level of SEPP1 in 34 pancreatic tissues was detected by qRT-PCR. (**E**) The immunoreactivity of SEPP1 in pancreatic tissues from 3 patients was assessed by IHC analysis. ***, *P* < 0.001

## Results

### SEPP1 is downregulated in pancreatic tumors, and it is associated with the abundance of CD8+ T cells

As shown in Fig. 1A, a total of 14 significantly down-regulated DEGs were identified between pancreatic tumors and their normal counterparts using GEO datasets (GSE183795, GSE19650, GSE125158 and GSE46234). Venn diagram was employed to compare DEGs identified among these datasets, and SEPP1 was identified as a downregulated gene in pancreatic tumors (Fig. 1B). The mRNA levels of these 14 DEGs were examined by qRT-PCR. As shown in Fig.S1A, a marked reduction of SEPP1 was observed in PC cell lines BxPC-3, PANC-1, CFPAC-1 and Capan-2 cells, compared with that in normal pancreatic duct epithelial cell lines H6C7 and hTERT-HPNE cells. SEPP1 with the most significant reduction in BxPC-3 and PANC-1 cells was selected for the subsequent studies. In addition, TIMER analysis revealed that SEPP1 expression was positively associated with the abundance of CD8^+^ T cells. In line with the bioinformatics analysis, qRT-PCR and IHC analysis confirmed the downregulation of SEPP1 in pancreatic tumors, compared with that in adjacent normal tissues (Fig. 1D-E). It is worth noting that SEPP1 was predominantly expressed in the cytoplasm of PC cells (Fig. 1E). Collectively, these findings suggest the decreased expression of SEPP1 in pancreatic tumors, and SEPP1 might play a critical role in CD8^+^ T cell-regulated immune responses.

**Fig. 2:**
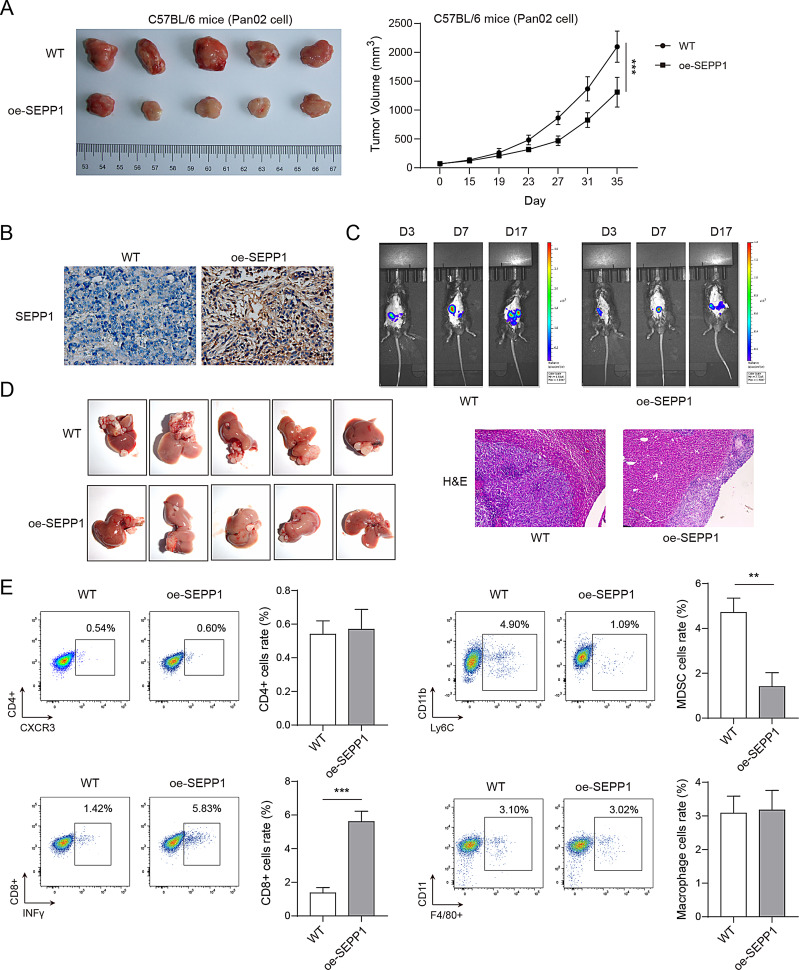
SEPP1 regulates immune escape of PC cells via modulating CD8^+^ T cell abundance. Pan02 cells (5 × 10^5^ cells) were injected into the flank of C57BL/6 mice subcutaneously. (**A**) Representative photos of xenograft tumors and tumor volumes. (**B**) The immunoreactivity of SEPP1 in xenograft tumors was assessed by IHC analysis. Pan02 cells (1 × 10^6^ cells) were injected into the tail vein of C57BL/6 mice. (**C**) The tumor growth and metastasis were monitored by in vivo bioluminescence imaging. (**D**) Representative photos and H&E staining of liver tissues. (**E**) The proportions of CD4 + T cells, CD8^+^ T cells, MDSCs and macrophages were detected by flow cytometry. **, *P* < 0.01; ***, *P* < 0.001. *n* = 5

### SEPP1 regulates immune escape of PC cells via modulating CD8+ T cell abundance

We next sought to investigate the biological role of SEPP1 in normal xenograft and liver metastasis mouse models. Control or SEPP1-overexpressing Pan02 cells were injected into C57BL/6 mice. As presented in Fig. 2A, overexpression of SEPP1 impaired tumor growth in vivo in which the tumor size and volume were remarkably decreased in SEPP1-overexpressing group. SEPP1 overexpression in xenograft tumors was confirmed by IHC analysis (Fig. 2B). Consistently, SEPP1 suppressed tumor growth and metastasis as detected by in vivo bioluminescence imaging (Fig. 2C). H&E staining showed that the numbers of liver metastases were dramatically reduced by SEPP1 overexpression (Fig. 2D). Intriguingly, the proportion of CD8^+^ T cells (CD8^+^/INF-γ^+^) was increased, while the abundance of myeloid-derived suppressor cells (MDSCs, CD11b^+^/Ly6C^+^) was decreased in SEPP1-overexpressing group, whereas there was no marked change of the abundance of CD4^+^ T cells (CD4^+^/CXCR3^+^) and macrophages (CD11b^+^/F4/80^+^) (Fig. 2E). In addition, overexpression of SEPP1 increased the proportion of NK cells (CD3^−^/CD49b^+^), while it had no effect on the percentage of neutrophil (CD15^+^/Ly-6G^+^), B cells (CD3^−^/CD19^+^) or dendritic cells (CD11c^+^/MHC II^+^) (Fig.S1B). These data indicate that SEPP1 regulates immune escape of PC cells, possibly via modulating the abundance of CD8^+^ T cells and MDSCs.

**Fig. 3:**
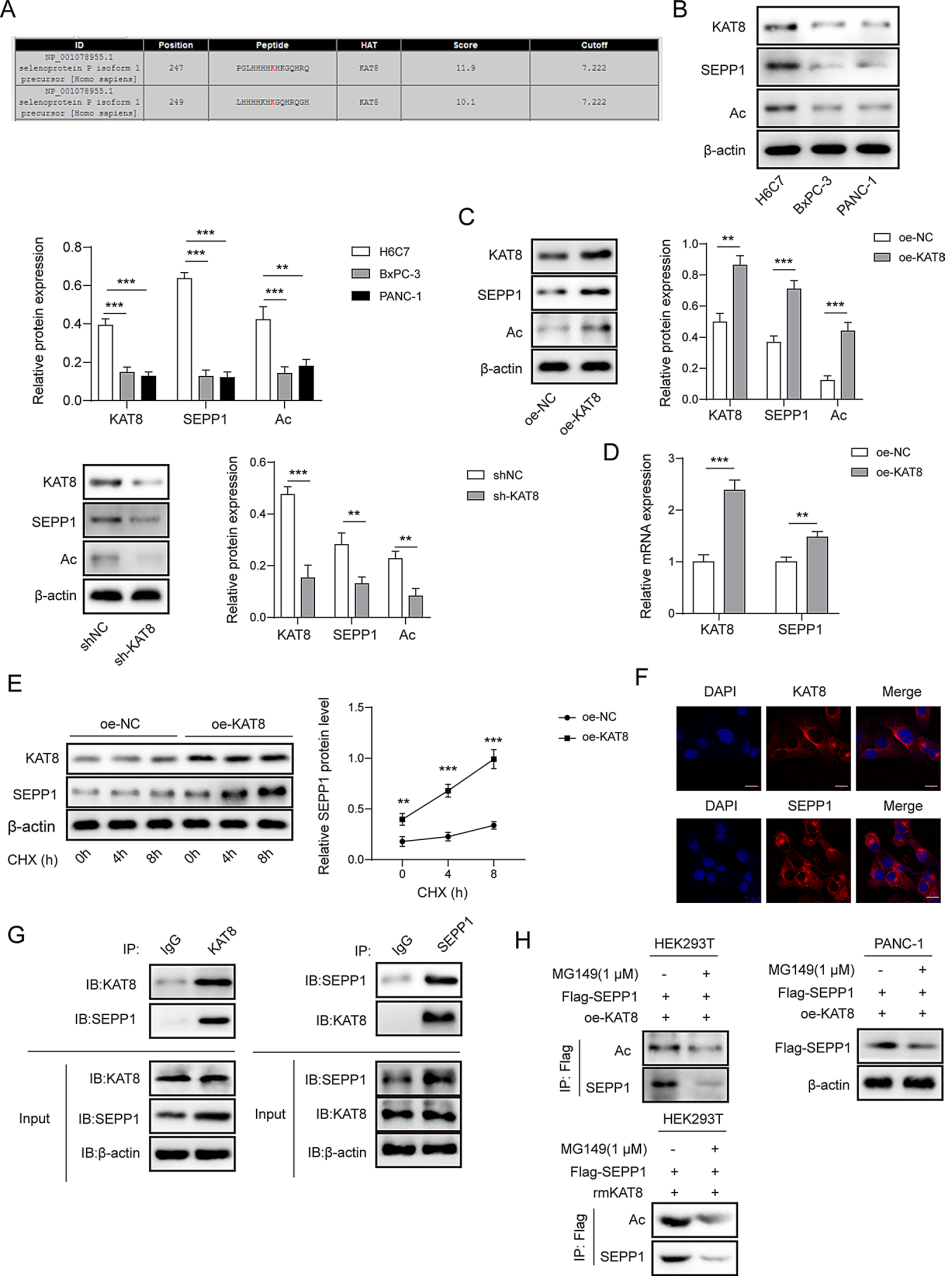
KAT8 positively regulates SEPP1 protein level through modulating lysine acetylation. (**A**) The lysine acetylation sites of SEPP1 were predicted using GPS-PAIL. (**B**) The protein levels of KAT8, SEPP1 and acetylation in H6C7, BxPC-3 and PANC-1 cells were detected by western blot. (**C**) The protein levels of KAT8, SEPP1 and acetylation in KAT8-overexpressing or KAT8-knockdown in HEK293T cells were detected by western blot. (**D**) The mRNA level of SEPP1 in HEK293T cells was detected by qRT-PCR. (**E**) The protein levels of KAT8 and SEPP1 in HEK293T cells were detected by western blot. (**F**) The subcellular localization of KAT8 or SEPP1 in HEK293T cells was detected by IF. (**G**) The direct interaction between KAT8 and SEPP1 in HEK293T cells was detected by co-IP. Whole cell lysates and normal IgG were used as input and negative control, respectively. (**H**) KAT8-mediated acetylation of SEPP1 in HEK293T or PANC-1 cells was detected by co-IP. **, *P* < 0.01; ***, *P* < 0.001. *n* = 3

### KAT8 positively regulates SEPP1 protein level through modulating lysine acetylation

GPS-PAIL predicted the acetylation sites of SEPP1 at lysine 247 and lysine 249 (K247/249), and KAT8 might catalyze the lysine acetylation of SEPP1 (Fig. 3A). Compared with H6C7 cells, KAT8 and SEPP1 expression, as well as SEPP1 acetylation level, were decreased in PC cell line BxPC-3 and PANC-1 cells (Fig. 3B). As expected, western blot showed that the SEPP1 acetylation levels were upregulated in KAT8-overexpressing HEK293T cells, while knockdown of KAT8 downregulated SEPP1 expression and acetylation (Fig. 3C), and the mRNA level of SEPP1 also was increased by KAT8 overexpression (Fig. 3D). It was speculated that the difference of SEEP1 expression might be a post-transcriptional modification. In addition, overexpression of KAT8 increased SEPP1 protein expression in the presence of protein synthesis inhibitor CHX time-dependently (Fig. 3E), suggesting that KAT8 regulates SEPP1 protein stability in HEK293T cells. IF staining further showed the cytoplasmic localization of KAT8 or SEPP1 in HEK293T cells (Fig. 3F). The direct interaction between KAT8 and SEPP1 was detected by co-IP in which either anti-KAT8 or anti-SEPP1 antibody successfully immunoprecipitated the other protein (Fig. 3G). In both HEK293T and PANC-1 cells, the HAT inhibitor MG149 inhibited KAT8-mediated acetylation of SEPP1, and the immunoprecipitated SEPP1 was decreased in the presence of MG149 (Fig. 3H). Similarly, KAT8 recombinant protein (rmKAT8) also mediated the acetylation of SEPP1 which was blocked by MG149 (Fig. 3H). These findings indicate that KAT8 mediates lysine acetylation of SEPP1, thus upregulating its protein level.

**Fig. 4:**
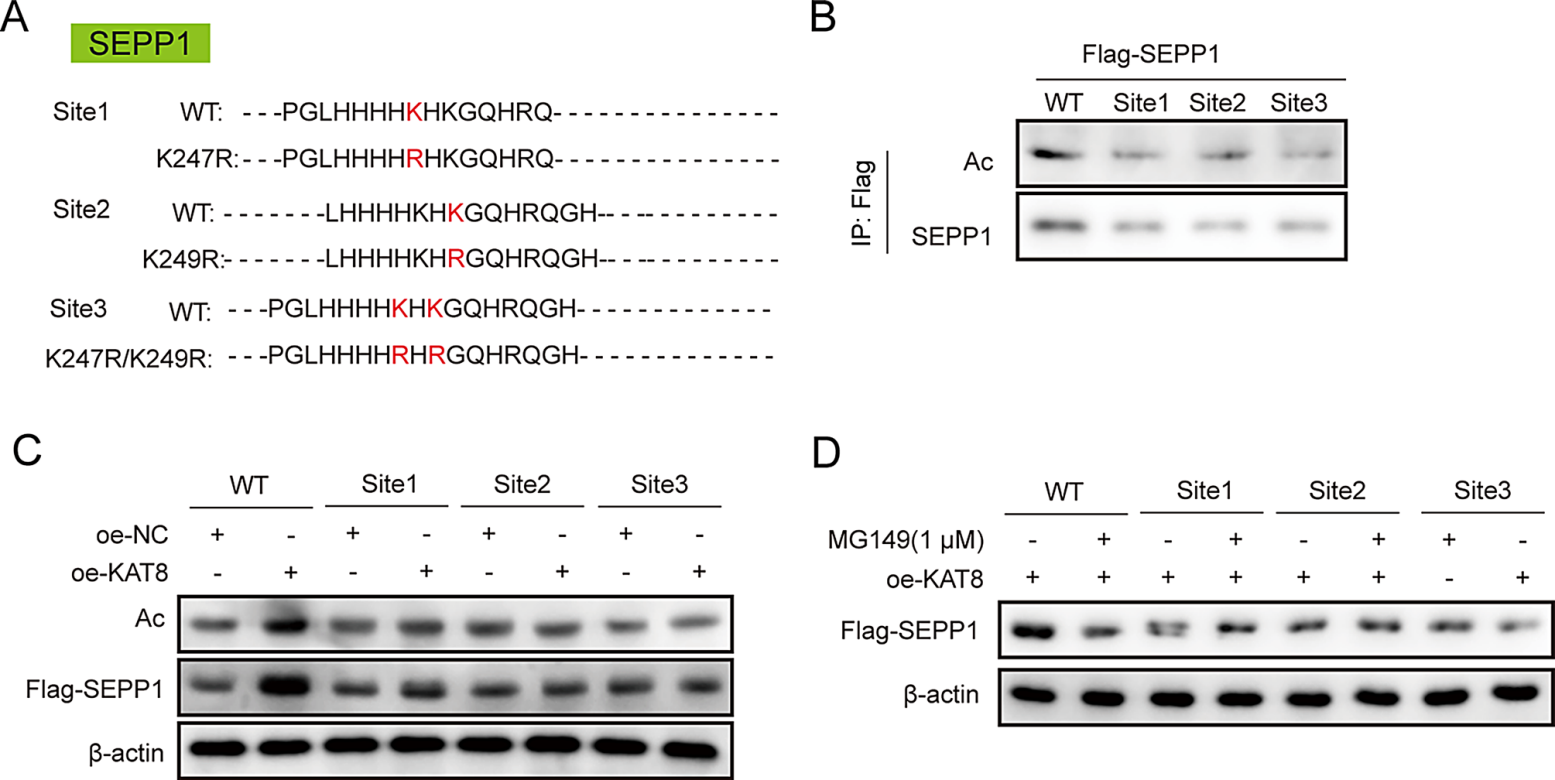
KAT8 catalyzes the acetylation of lysine 247/249 on SEPP1. (**A**) The K-to-R mutations of K247/249. (**B**) The acetylation and expression of SEPP1 in HEK293T cells were detected by co-IP. (**C-D**) KAT8-regulated SEPP1 acetylation and expression in HEK293T cells was detected by co-IP. *n* = 3

**Fig. 5:**
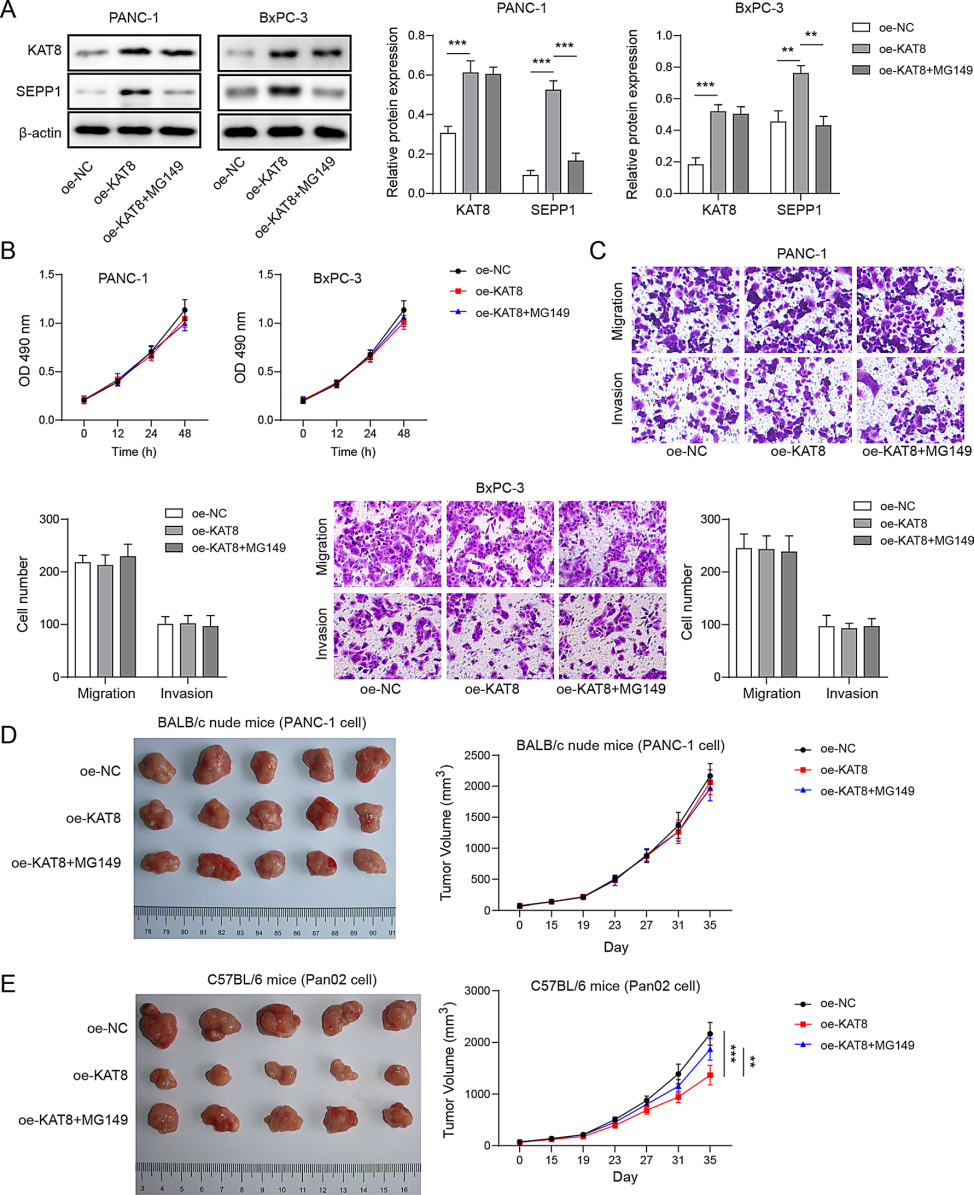
KAT8 suppressed PC progression via modulating SEPP1-dependent immune responses. (**A**) The protein levels of KAT8 and SEPP1 were detected by western blot in BxPC-3 and PANC-1 cells. (**B**) Cell viability was monitored by CCK-8 assay in BxPC-3 and PANC-1 cells. (**C**) BxPC-3 and PANC-1 cells migration and invasion were assessed by Transwell migration and invasion assays. *n* = 3. PANC-1 cells (4 × 10^6^ cells) were inoculated into the right axillary fossa of BALB/c nude mice. Pan02 cells (5 × 10^5^ cells) were injected into the flank of C57BL/6 mice subcutaneously. Representative photos of xenograft tumors and tumor volumes in nude mouse xenograft model (**D**) or normal mouse xenograft model (**E**). *n* = 5. **, *P* < 0.01; ***, *P* < 0.001

### KAT8 catalyzes the acetylation of lysine 247/249 on SEPP1

To further test if K247/249 was implicated in KAT8-mediated acetylation of SEPP1, K-to-R mutants of K247R or/and K249R were generated, namely site1-MUT, site2-MUT and site3-MUT (K247R/K249R double mutation) (Fig. 4A). As anticipated, overexpression study revealed that the acetylation and expression of SEPP1 were decreased in site1-MUT, site2-MUT and site3-MUT groups, compared with WT group (Fig. 4B). Moreover, K247R/K249R double mutant induced more prominent reduction of SEPP1 acetylation (Fig. 4B). Mutation of K247R, K249R or K247R/K249R abolished KAT8-mediated acetylation and upregulation of SEPP1 in HEK293T cells (Fig. 4C). Additionally, the negative effects of MG149 on KAT8-catalyzed SEPP1, as well as on KAT8-increased SEPP1 expression, were also abrogated by the mutation of K247R, K249R or K247R/K249R (Fig. 4D). It is worth noting that the mutation of K362R (site4-MUT) or K366R (site5-MUT) had no significant effect on the acetylation status and expression of SEPP1, as well as KAT8-induced SEPP1 acetylation and expression (Fig. S3). These data suggest that KAT8 catalyzes the acetylation of K247/249 on SEPP1.

**Fig. 6:**
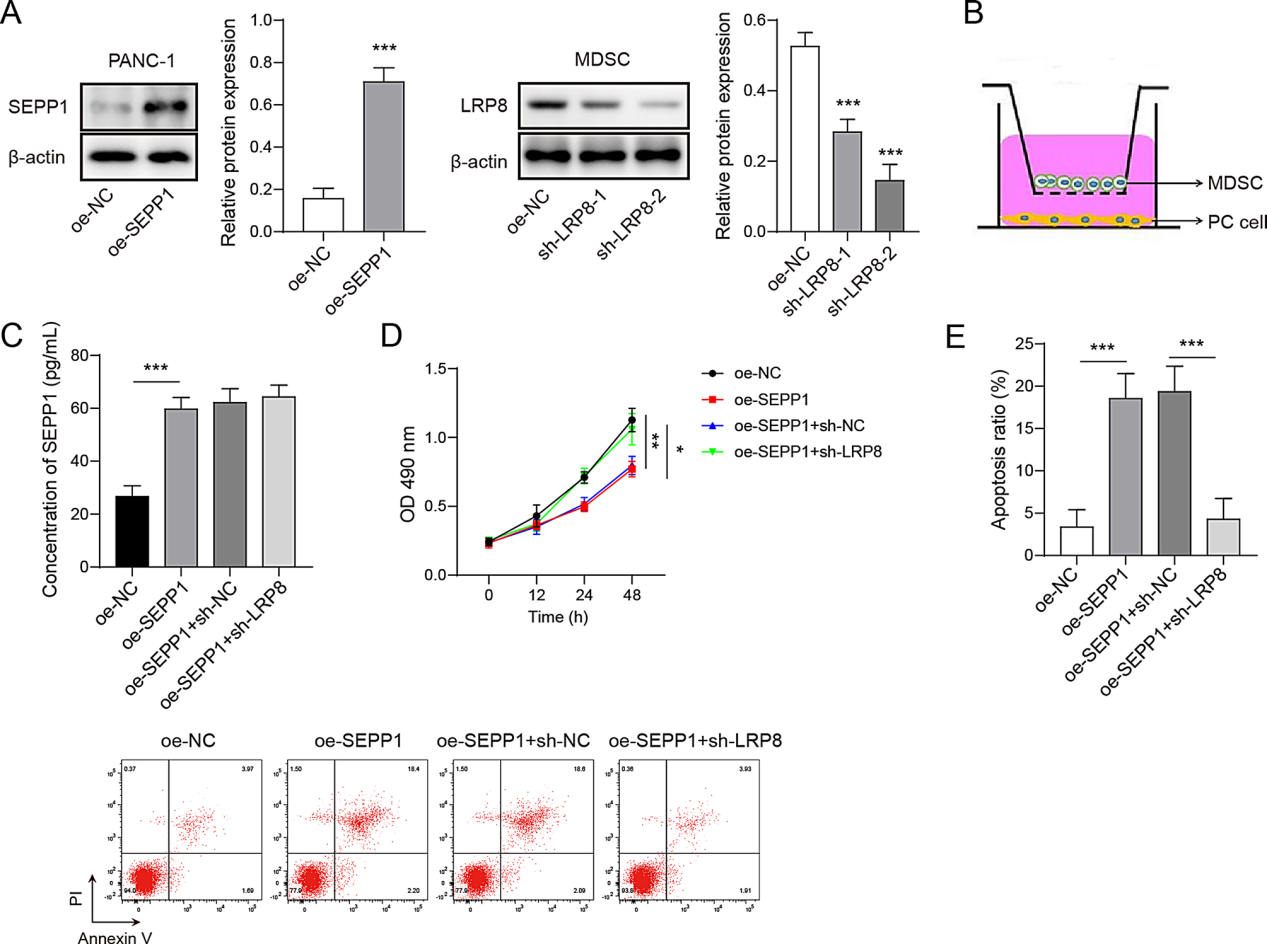
SEPP1 promotes the viability of MDSCs in a LRP8-dependent manner. (**A**) The protein levels of SEPP1 in PANC-1 cells and LRP8 in MDSCs were detected by western blot. (**B**) The diagram of co-culture system. (**C**) The secretion of SEPP1 was measured by ELISA assay. (**D**) Cell viability was monitored by CCK-8 assay. (**E**) Cell apoptosis was detected by Annexin V-FITC/PI staining with quantitative analysis. ***, *P* < 0.001. *n* = 3

**Fig. 7:**
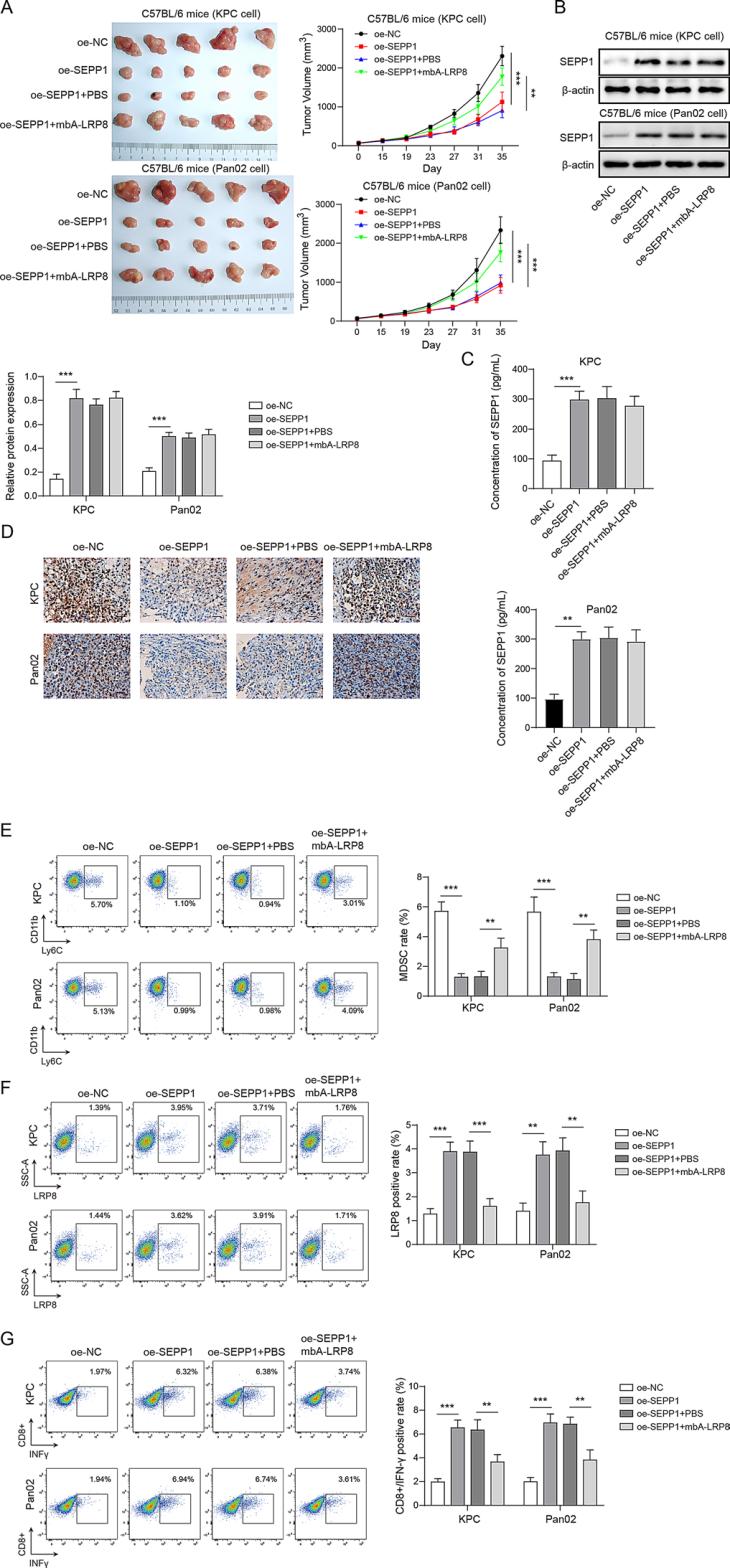
SEPP1 regulates CD8^+^ T cell-mediated immune responses via MDSCs. KPC and Pan02 cells (5 × 10^5^ cells) were injected into the flank of C57BL/6 mice subcutaneously. (**A**) Representative photos of xenograft tumors and tumor volumes in normal mouse xenograft model. (**B**) The protein level of SEPP1 was detected by western blot. (**C**) The serum level of SEPP1 in mice was detected by ELISA assay. (**D**) The immunoreactivity of Ki-67 was detected by IHC analysis. (**E**) The proportion of MDSCs was detected by flow cytometry. (**F**) The expression of LRP8 in MDSCs was analyzed by flow cytometry. (**G**) The proportion of CD8^+^ T cells and IFN-γ expression were detected by flow cytometry. **, *P* < 0.01; ***, *P* < 0.001. *n* = 5

**Fig. 8:**
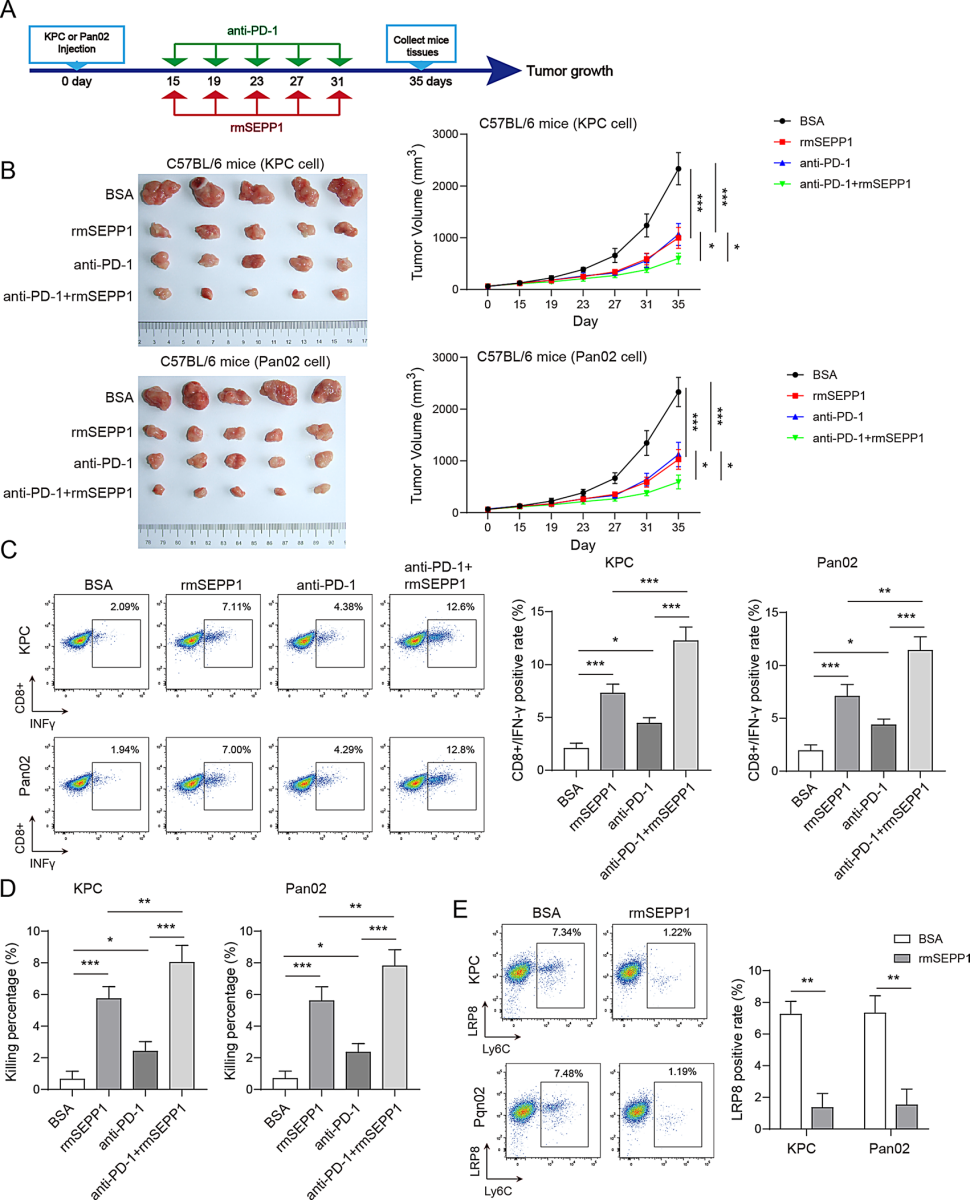
SEPP1 recombinant protein enhances the efficacy of anti-PD-1 therapy in PC mouse model. KPC and Pan02 cells (5 × 10^5^ cells) were injected into the flank of C57BL/6 mice subcutaneously. (**A**) The scheme of the in vivo experiment design. (**B**) Representative photos of xenograft tumors and tumor volumes in normal mouse xenograft model. Pan02 cells (1 × 10^6^ cells) were injected into the tail vein of C57BL/6 mice. (**C**) The proportion of CD8^+^/IFN-γ^+^ T cells was analyzed by flow cytometry. (**D**) The killing efficacy of CD8^+^ T cells was detected by CTL. (**E**) The number of MDSCs was analyzed by flow cytometry. *, *P* < 0.05; **, *P* < 0.01; ***, *P* < 0.001. *n* = 5

### KAT8 regulates immune responses to suppress PC progression via modulating SEPP1 expression

To further delineate the function of KAT8/SEPP1 axis, overexpression studies were conducted in PANC-1 and BxPC-3 cells. Western blot showed that KAT8 overexpression upregulated SEPP1 expression, while this effect was attenuated by MG149 in both PC cells (Fig. 5A). MG149 caused no change of KAT8 expression (Fig. 5A). CCK-8 and transwell assays revealed that OE-KAT8 or OE-KAT8 + MG149 had no remarkable effect on cell proliferation, migration and invasion in PANC-1 and BxPC-3 cells (Fig. 5B-C). Considering the immunodeficiency of nude mice, two different mouse models were used for xenograft study. Notably, OE-KAT8 or OE-KAT8 + MG149 exhibited no effect on tumor growth in nude mouse xenograft model (Fig. 5D). By contrast, overexpression of KAT8 dramatically inhibited tumor growth, and the suppressive effect was abolished by MG149 in normal C57BL/6 mouse xenograft model (Fig. 5E). Moreover, C57BL/6 tumor-bearing mice were treated with LXRβ agonist RGX-104 or GW3965 which induces MDSCs depletion [18]. As presented in Fig. S4A, RGX-104 or GW3965 suppressed tumor growth in vivo. Flow cytometry further revealed that RGX-104 or GW3965 successfully induced MDSCs depletion in xenograft tumors, and lack of MDSCs increased the population of CTLs (Fig. S4B-C). Furthermore, KAT8 overexpression decreased the number of MDSCs, while MG149 protected against this negative effect as detected by flow cytometry (Fig. S5A). By contrast, overexpression of KAT8 increased the population of CTLs, whereas MG149 decreased the percentage of CTLs in xenograft tumors (Fig. S5B). As presented in Fig. S5C, overexpression of KAT8 successfully induced KAT8 expression, along with the upregulation of SEPP1. On the contrary, transfection of sh-SEPP1 reduced SEPP1 expression, but had no effect on KAT8 protein level in Pan02 cells (Fig. S5C). CCK-8 and transwell assays revealed that KAT8 overexpression or SEPP1 knockdown had no significant effect on cell proliferation, migration and invasion in Pan02 cells. The transfected Pan02 cells were injected into C57BL/6 mice. Xenograft study showed that overexpression of KAT8 suppressed tumor growth, whereas silencing of SEPP1 attenuated the tumor suppressive effect of KAT8 in vivo (Fig. S5F). These findings indicate that KAT8 regulates immune responses to suppress PC progression, possibly via modulating SEPP1 expression.

### SEPP1 impairs the viability of MDSCs in a LRP8-dependent manner

We next sought to test whether SEPP1 interacted with its receptor LRP8 on MDSCs. As shown in Fig. 6A and S1A, SEPP1 overexpression successfully upregulated SEPP1 expression in PANC-1 and BxPC-3 cells, and transfection of sh-LRP8-2 markedly downregulated LRP8 level in MDSCs. Sh-LRP8-2 was thus selected for the subsequent experiments. For co-culture system, transfected PC cells and MDSCs were cultured in the lower and upper chambers, respectively (Fig. 6B). ELISA assay revealed that the secretion of SEPP1 was elevated in SEPP1-overexpressing group, while LRP8 knockdown exhibited no effect on SEPP1 secretion (Fig. 6C). CCK-8 assay revealed that SEPP1 overexpression in PANC-1 and BxPC-3 cells inhibited the proliferation of MDSCs, whereas this effect was abrogated by LRP8 knockdown (Fig. 6D and S1C). Moreover, SEPP1-enhanced cell apoptosis was counteracted by sh-LRP8 in MDSCs as detected by Annexin V-FITC/PI staining (Fig. 6E and S1E). These findings suggest that SEPP1 impairs the viability of MDSCs in a LRP8-dependent manner.

### SEPP1 regulates CD8+ T cell-mediated immune responses via MDSCs

To further unravel the mechanism underlying SEPP1-dependent immune responses, normal C57BL/6 xenograft mice were randomly divided into four groups: OE-NC, OE-SEPP1, OE-SEPP1 + BSA and OE-SEPP1 + mAb-LRP8. As presented in Fig. 7A, overexpression of SEPP1 inhibited tumor growth, while this negative effect was reverse by antibody against LRP8. The elevation of SEPP1 in SEPP1-overexpressing groups was confirmed by western blot, and the injection of mAb-LRP8 had no effect on SEPP1 expression in both KPC and Pan02 cells (Fig. 7B). ELISA assay showed that overexpression of SEPP1 increased the serum level of SEPP1 in mice, whereas the addition of mAb-LRP8 had no effect on SEPP1 serum level (Fig. 7C). Consistently, IHC analysis showed the downregulation of tumor proliferation marker Ki-67 in xenograft tumors derived from SEPP1-overexpressing cells, while mAb-LRB8 exerted a rescue effect on Ki-67 expression (Fig. 7D). Flow cytometry further revealed that SEPP1 overexpression in PC cells decreased the population of MDSCs, while antibody against LRP8 exerted a rescue effect (Fig. 7E). In addition, overexpression of SEPP1 upregulated LRP8 level in MDSCs (Fig. 7F), and this was accompanied with the increased abundance of CD8^+^ T cells and elevated IFN-γ expression (Fig. 7G). In addition, mAb-LRP8 neutralized LRP8 expression in MDSCs (Fig. 7F), and it also downregulated the abundance of CD8^+^ T cells and IFN-γ expression (Fig. 7G). Collectively, these data indicate that SEPP1 interacts with LRP8 on MDSCs, and contributes to CD8^+^ T cell-mediated immune responses. Furthermore, in vivo findings also showed that rmSEPP1 injection had no effect on RGX-104-suppressed tumor growth (Fig. S4D). Flow cytometry and CTL assay revealed that RGX-104-mediated MDSCs depletion increased the proportion of CD8^+^ T cells and enhanced the killing efficiency of CD8^+^ T cells, while rmSEPP1 exerted no remarkable effect on CD8^+^ T cell percentage or the killing efficiency of CD8^+^ T cells in the absence of MDSCs (Fig. S4E-F).

### SEPP1 recombinant protein enhances the efficacy of anti-PD-1 therapy in PC mouse model

For implantation of xenograft tumor, KPC or Pan02 cells were injected into the right axillary fossa of normal mice. From day 15 to 31 post-inoculation, mouse SEPP1 recombinant protein (rmSEPP1) or/and anti-PD-1 antibody were injected every four days. Mice were sacrificed and the xenograft tumors were dissected on day 35 for subsequent analysis (Fig. 8A). Similar with SEPP1 overexpression, rmSEPP1 suppressed tumor growth, and it also potentiated the negative effect of anti-PD-1 antibody on tumor growth (Fig. 8B). Notably, rmSEPP1 or anti-PD-1 antibody increased the abundance of CD8^+^/IFNγ^+^ T cells, and much more CD8^+^/IFNγ^+^ T cells were detected in anti-PD-1 + rmSEPP1 group (Fig. 8C). CTL assay further showed that rmSEPP1 greatly enhanced the killing efficiency of CD8^+^ T cells, and an increased killing efficiency of CD8^+^ T cells was also found in anti-PD-1 + rmSEPP1 group (Fig. 8D). Furthermore, flow cytometry showed that rmSEPP1 reduced the number of MDSCs (Fig. 8E). It is worth noting that the injection of rmSEPP1 significantly increased SEPP1 expression in xenograft tumors (Fig. S2A). By contrast, rmSEPP1 had no effect on SEPP1 level in heart, liver, spleen, lung and kidney (Fig.S2B). These findings indicate that rmSEPP1 preferentially bound to tumors rather than normal tissues, including heart, liver, spleen, lung and kidney. H&E staining also showed that rmSEPP1 caused no histological change of these tissues (Fig.S2 A-C). Together, these data suggest that rmSEPP1 improves the efficacy of anti-PD-1 therapy in PC mouse model.

## Discussion

Despite the advance in systemic chemotherapy, the clinical outcomes of PC remain poor. Notably, an immune-suppressive TME, including MDSCs, tumor-associated macrophages and T regulatory cells (Tregs), has been reported in PC which leads to immune escape from anti-tumor immune system [3, 23]. It is of interest to identify the novel biomarker responsible for the immune suppression. In consistent with the previous findings in different cancers, SEPP1 was also downregulated in PC [11–15]. While an early study has demonstrated that the expression of SEPP1 is higher in gemcitabine-resistant PC cell line KLM1 cells, compared with the parental cells. It has been reported that SEPP1 decreases the chemosensitivity to gemcitabine by reducing ROS level [24], suggesting that the biological function of SEPP1 might be cell-type dependent. In addition to its antioxidant role, we reported that SEPP1 expression positively correlated with CD8^+^ T cell abundance in this study. In vivo studies further showed the tumor suppressive effects of SEPP1 on tumor growth and liver metastasis which was accompanied with increased number of CD8^+^ T cell and reduced MDSCs population. MDSCs are immunosuppressive cells comprised of dendritic cells (DCs), granulocytes and immature monocytes [25]. MDSCs inhibits proliferation and triggers apoptosis of CD8^+^ T cells, and its abundance negatively correlated with the infiltration of CD8^+^ T cells [26]. SEPP1-mediated changes of CD8^+^ T cells and MDSCs indicate its potential role in immune suppression in PC. Immune microenvironment is a highly complex system [27]. This study mainly focused on the effects of SEPP1 on CD8^+^ T cells and MDSCs in the immune microenvironment. The role of SEPP1 on the other immune cells, especially NK cells, required further investigation. It is worth noting that qRT-PCR also confirmed the reduction of STEAP4, LINC00671, CD36 or SPERPINI2 in PC cells. The functions of these DEGs merit in-depth investigation in the future study.

Recent studies have illustrated the crucial role of histone acetylation regulator-mediated TME infiltration in HCC and lung adenocarcinoma [28, 29]. Previous studies have reported that KAT8 acetylates histone H4 at K16, as well as Nrf2 and p53 [30]. Dysregulation of KAT8 is associated with the progression of various cancers [31]. Interestingly, KAT8 was predicted as a HAT catalyzed the acetylation of SEPP1 at K247/249 in this study. KAT8 upregulated SEPP1 protein expression in the presence of CHX, suggesting that KAT8 positively regulated SEPP1 expression via protein acetylation. KA8-mediated acetylation on SEPP1 enhanced the protein stability of SEPP1, thereby facilitating the binding between SEPP1 and its receptor LRP8. This finding was further confirmed by using MG149 in which this HAT inhibitor suppressed KAT8-mediated acetylation of SEPP1, thus reducing its protein level. Mutational study indicates that either K247 or K249 is required for KAT8-catalyzed acetylation of SEPP1, instead of K362 or K366. It has been illustrated that KAT8 is widely expressed in immune cells, and it mediates the acetylation of IRF3 at K359 to suppress antiviral immunity [32]. Similarly, in vitro studies further revealed that KAT8 or MG149 had no effect on the proliferative or metastatic properties of PC cells, as well as in immunodeficient nude mice, indicating that KAT8-catalyzed acetylation regulates immune responses to suppress PC. Overexpression of KAT8 increased SEPP1 mRNA level as detected by qRT-PCR. Previous reports have demonstrated that KAT8 regulates gene expression via modulating acetylation of lysine on histone [33, 34]. Subsequent studies also illustrated that a wide range of non-histone proteins are involved in transcriptional regulation after lysine acetylation by KAT8 [35, 36]. We thus supposed that different regulatory mechanisms might be implicated in KAT8-mediated regulation of SEPP1. The crosstalks among these mechanisms require in-depth investigation in the future study. Furthermore, our preliminary bioinformatics analysis predicted various post-translational modifications of SEPP1, including promoter methylation, ubiquitination and acetylation, etc. However, no CpG island was found in the promoter region of SEPP1. MARCH family protein was predicted as a potential E3 ubiquitin ligase responsible for SEPP1 ubiquitination, while no change of MARCH family protein expression was observed in PC cells. By contrast, the prediction score of acetylation was high, and KAT8 was differentially expressed in PC cells. This study thus focused on the acetylation of SEPP1 in PC. SEPP1 might also be regulated by the other post-translational modifications. Our research group is continuing to conduct in-depth research on SEPP1 modification in the future study.

It is well-established that MDSCs serve as key players in the immune-suppressive TME of PC. Depletion of MDSCs induces the accumulation of activated CD8^+^ T cells in TME, and triggers apoptosis of PC cells [37]. In accordance with these findings, overexpression of SEPP1 in PC cells impaired the viability of MDSCs, while this effect was abrogated in MDSCs lack of LRP8, suggesting the pivotal role of LRP8 in SEPP1-suppressed MDSCs viability. These findings were validated by in vivo experiments in which SEPP1-inhibited tumor growth, as well as SEPP1-increased CD8^+^ T cell abundance and IFN-γ expression, were counteracted by anti-LRP8 antibody. This is consistent with a previous report which has demonstrated that LXR/ApoE axis represses MDSCs viability in dependent of LRP8, thereby activating CD8^+^ T cells and facilitating anti-tumor immunity [18]. Interestingly, co-treatment of RGX-104 and rmSEPP1 showed that rmSEPP1 could not enhance the function of CD8^+^ T cells in the absence of MDSCs, suggesting that SEPP1 regulates the percentage and killing efficiency of CD8^+^ T cells in a MDSCs-dependent manner.

Emerging evidence supports that the immune checkpoint molecule PD-1 on immune cells interacted with its receptor PD-L1 on tumor cells, macrophages and DCs [38]. Anti-PD-1 therapy improves the clinical outcomes of various cancers [38, 39]. However, PC is recognized as an immune-quiescent cancer, and the beneficial effects of anti-PD-1 therapy for PC remain limited [23]. In recent years, researchers have demonstrated the advancement of combination therapy with anti-PD-1 therapy in PC [40–42]. For instance, Fc-optimized antibody against CD25 improves the efficacy of anti-PD-1 therapy in established tumors via depleting Tregs in TME [42]. Similarly, we found that rmSEPP1 remarkably improved the efficacy of anti-PD-1 therapy through activating CD8^+^ T cells and decreasing LRP8 expression in MDSCs. These findings identified a promising combination therapy for PC.

In conclusion, we reported that KAT8 catalyzed the acetylation of SEPP1 at K247/249. SEPP1 impaired MDSCs viability via LRP8, thereby activating CD8^+^ T cells to promote anti-tumor immunity in PC. SEPP1 recombinant protein augmented the efficacy of anti-PD-1 therapy in PC model, providing novel insights into the combination therapy for PC.

## Electronic supplementary material

Below is the link to the electronic supplementary material.

Supplementary Material 1

## Data Availability

All data generated or analyzed during this study are included in this article. The datasets used and/or analyzed during the current study are available from the corresponding author on reasonable request.

## Abbreviations

CHX: cycloheximide
CTLs: cytotoxic T lymphocytes
DCs: dendritic cells
DEGs: differentially expressed genes
HAT: histone acetyltransferase
H&E: hematoxylin and eosin
IF: immunofluorescence
IHC: immunohistochemistry
LRP8: low-density lipoprotein receptor-related protein 8
MDSCs: myeloid-derived suppressor cells
PC: pancreatic cancer
PD-1: programmed cell death-1
PFA: paraformaldehyde
ROS: reactive oxygen species
SEPP1: selenoprotein P
TIMER: tumor immune estimation resource
TME: tumor microenvironment
Tregs: T regulatory cells
