# Supplementary material for: KAT8 catalyzes the acetylation of SEPP1 at lysine 247/249 and modulates the activity of CD8+ T cells via LRP8 to promote anti-tumor immunity in pancreatic cancer
Source: Cell Biosci. 2025 Feb 19;15:24. doi: 10.1186/s13578-025-01356-3 (PMC11841300; doi:10.1186/s13578-025-01356-3)
Supplement: Supplementary file 1 — Supplementary Material 1 [file 13578_2025_1356_MOESM1_ESM.docx]

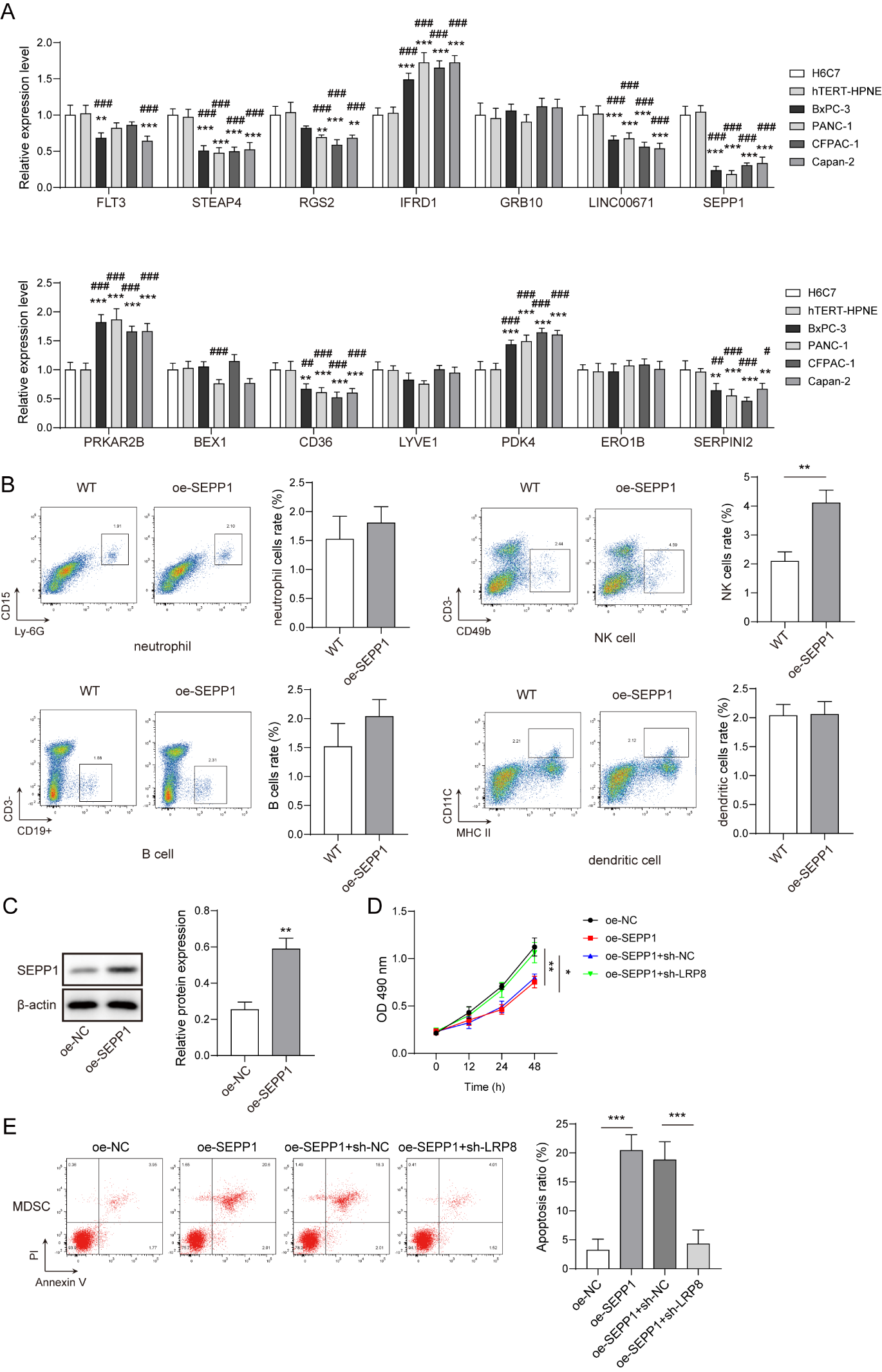


**Figure S1. SEPP1 promotes the viability of MDSCs in a LRP8-dependent manner.** (A) The mRNA levels of 14 DEGs in H6C7, hTERT-HPNE, BxPC-3, PANC-1, CFPAC-1 and Capan-2 cells were detected by qRT-PCR. Compared with the H6C7 group, **, P < 0.01; ***, P < 0.001. Compared with the hTERT-HPNE group, ^#^, P < 0.05; ^##^, P < 0.01; ^###^, P < 0.001. n = 3. Pan02 cells (1 × 10^6^ cells) were injected into the tail vein of C57BL/6 mice. (B) The proportions of neutrophil, NK, B and dendritic cells were detected by flow cytometry. n = 5. BxPC-3 cells were co-cultured with MDSCs. (C) The protein level of SEPP1 in BxPC-3 cells was detected by western blot. (D) Cell viability was monitored by CCK-8 assay. (E) Cell apoptosis was detected by Annexin V-FITC/PI staining with quantitative analysis. n = 3. *, P < 0.05; **, P < 0.01; ***, P < 0.001.


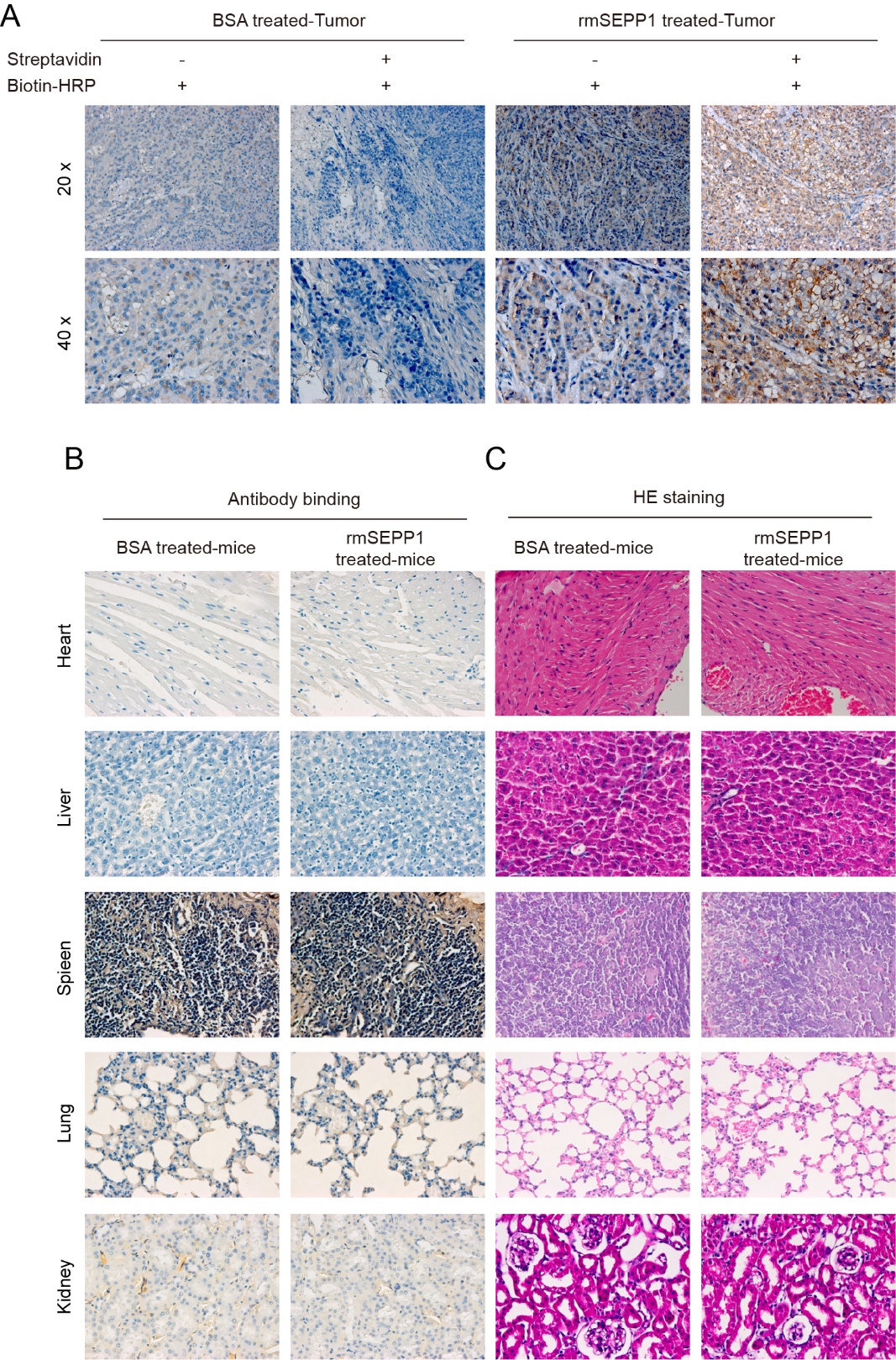


**Figure S2. SEPP1 recombinant protein enhances the efficacy of anti-PD-1 therapy in PC mouse model.** Pan02 cells (1 × 10^6^ cells) were injected into the tail vein of C57BL/6 mice. Normal xenograft mice were treated with biotinylated rmSEPP1 or BSA control. Signal was detected by streptavidin-HRP. (A) IHC analysis of rmSEPP1 in xenograft tumors. (B) IHC analysis of rmSEPP1 in heart, liver, spleen, lung and kidney tissues. (C) Histological changes of heart, liver, spleen, lung and kidney tissues were detected by H&E staining. n = 5.


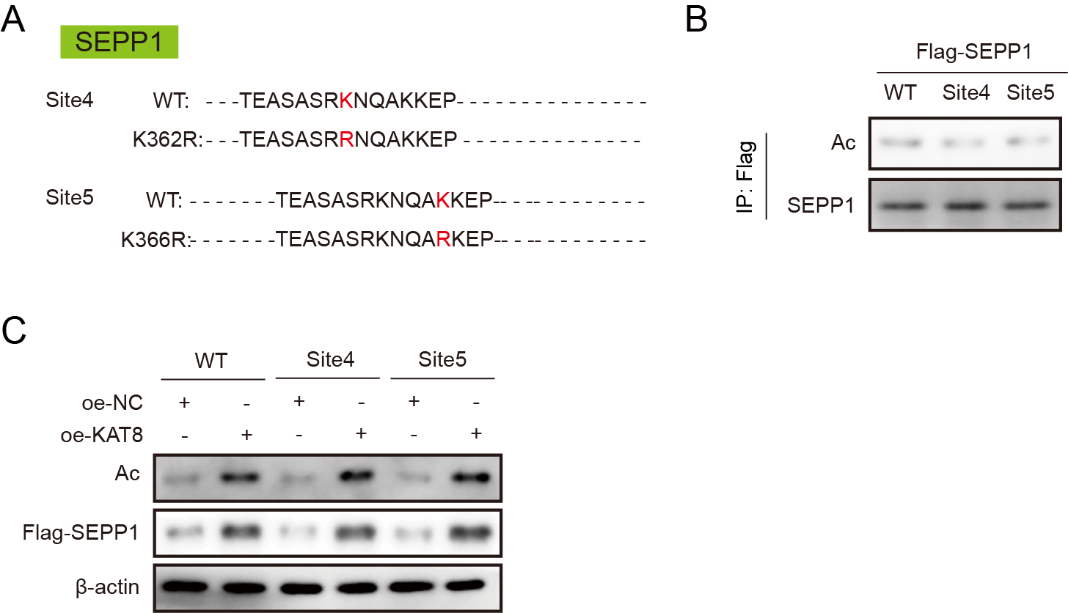


**Figure S3. The lysine 362/366 on SEPP1 has no effect on KAT8-induced SEPP1 expression and acetylation.** (A) The K-to-R mutations of K362/366. (B) The acetylation and expression of SEPP1 in HEK293T cells were detected by co-IP. (C) KAT8-regulated SEPP1 expression and acetylation in HEK293T cells was detected by co-IP. n = 3.


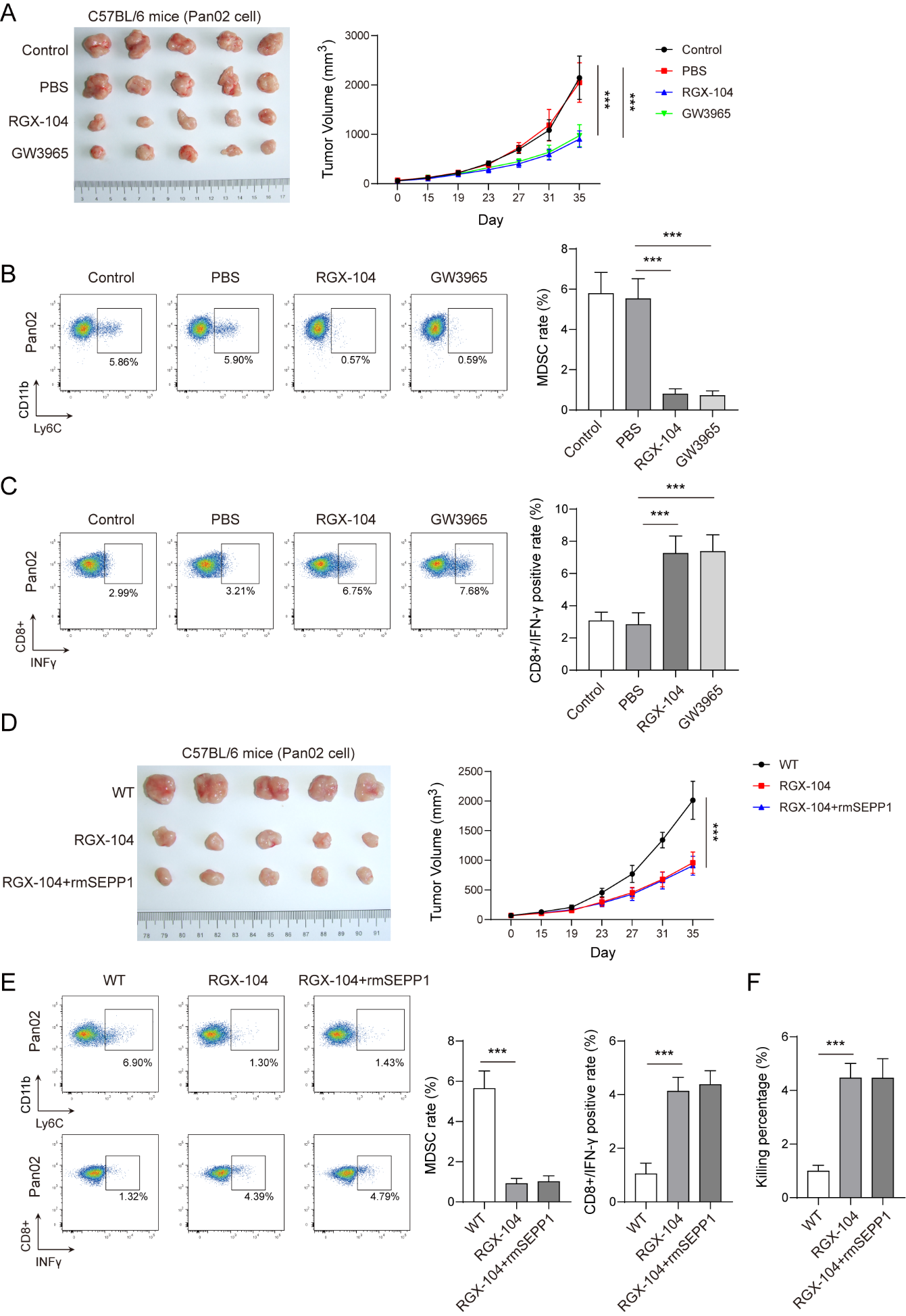


**Figure S4. MDSCs are implicated in the regulation of PC progression.** Pan02 cells (5 × 10^5^ cells) were injected into the flank of C57BL/6 mice subcutaneously. (A) Representative photos of xenograft tumors and tumor volumes. Pan02 cells (1 × 10^6^ cells) were injected into the tail vein of C57BL/6 mice. (B-C) The proportions of MDSCs and CD8^+^ T cells were detected by flow cytometry. (D) The photos of xenograft tumors and tumor volumes in normal mouse xenograft model. (E) The proportions of MDSCs and CD8^+^ T cells were detected by flow cytometry. (F) The killing efficacy of CD8^+^ T cells was detected by CTL. n = 5. ***, P < 0.001.


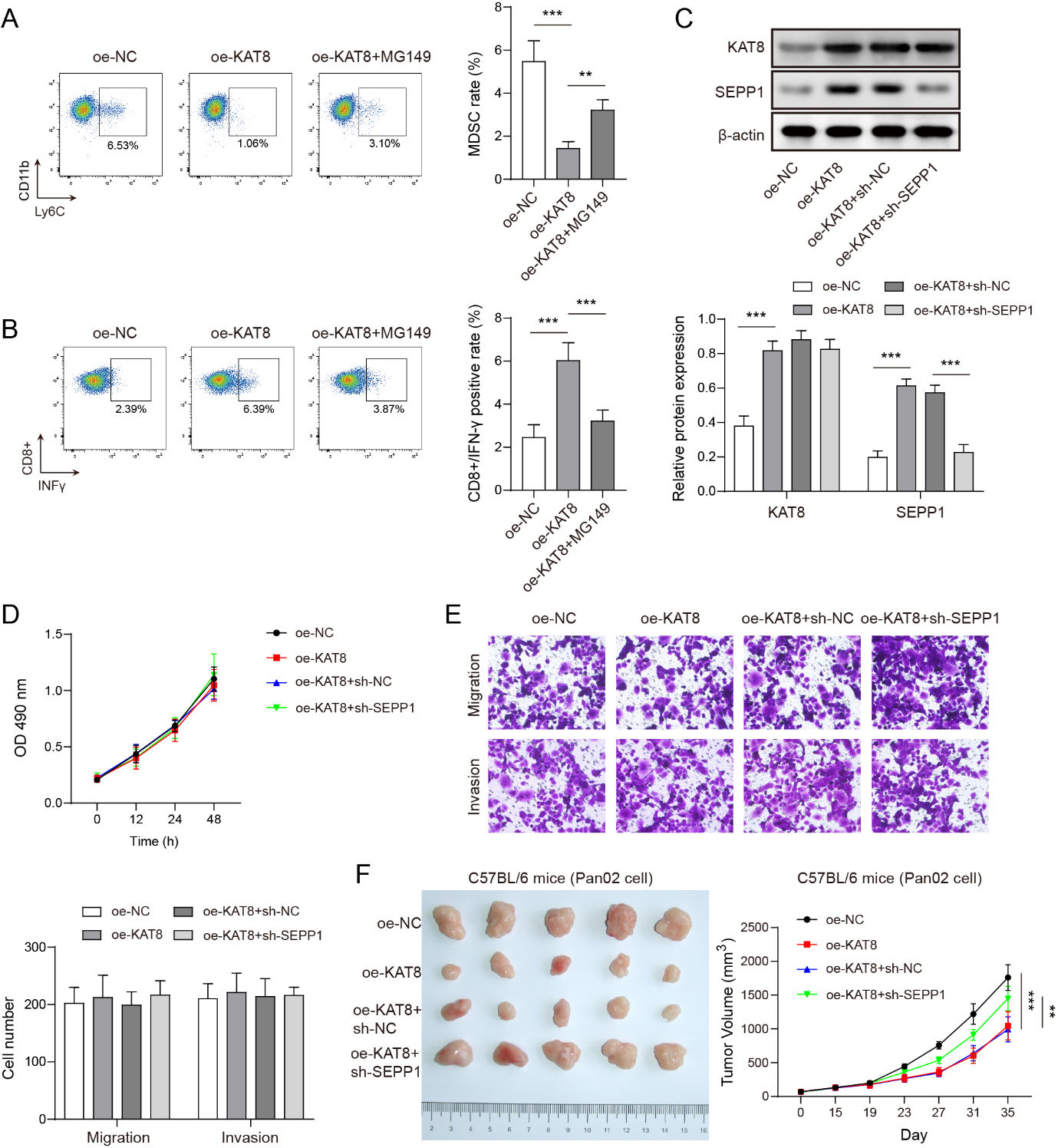


**Figure S5. KAT8 regulates the proportions of MDSCs and CTLs.** Pan02 cells (1 × 10^6^ cells) were injected into the tail vein of C57BL/6 mice. (A) The proportion of MDSCs was detected by flow cytometry. (B) The proportion of CD8^+^ T cells was detected by flow cytometry. n = 5. (C) The protein levels of KAT8 and SEPP1 were detected by western blot in PANC-1 cell. (D) PANC-1 cell viability was monitored by CCK-8 assay. (E) PANC-1 cell migration and invasion were assessed by Transwell migration and invasion assays. n = 3. (F) Pan02 cells (5 × 10^5^ cells) were injected into the flank of C57BL/6 mice subcutaneously. The photos of xenograft tumors and tumor volumes in normal mouse xenograft model. n = 5. **, P < 0.01; ***, P < 0.001.
